# Current concepts for oil decontamination of crush injuries: a review

**DOI:** 10.1186/1754-9493-8-22

**Published:** 2014-05-13

**Authors:** Chante Karimkhani, Mahsa Amir, Robert P Dellavalle, Kyros Ipaktchi

**Affiliations:** 1 Columbia University College of Physicians and Surgeons, 630 W. 168th Street, New York, NY 10032, USA; 2 Department of Dermatology, University of Colorado School of Medicine, Anschutz Campus, Aurora, CO 80045, USA; 3 Dermatology Service, Department of Veterans Affairs Medical Center, 1055 Clermont Street, Box 165, Denver, CO, USA; 4 Department of Epidemiology, Colorado School of Public Health, Aurora, CO 80045, USA; 5 Department of Orthopaedic Surgery, Denver Health Medical Center, 777 Bannock Street, Denver, CO 80204, USA

**Keywords:** Dermatology, Cutaneous wound, Crush injuries, Petroleum, Skin decontamination, Like dissolves like, Lipophilic, Solvent

## Abstract

This anecdotal, non-systematic review serves to explore the principles and methods of effective oil decontamination from cutaneous wounds, particularly crush injuries. The current expansion of the petroleum industry is necessary to meet increasing world demands for oil. Most stages of oil refining and applications involve significant injury risks, particularly for crush injuries that become contaminated with petroleum compounds. A literature review regarding a standard of care for effective cutaneous oil decontamination is lacking. Based on case reports, animal models, and in vitro studies identified in our expert opinion review, standard water and soap cleansing may not be an appropriate approach. Instead, the principle of ‘like dissolves like’ guides the use of lipophilic, petroleum-derived solvents to attract and subsequently dissolve the petroleum contaminant from the skin injury. Limitations include paucity of and dated literature sources regarding the topic as well as no models specifically addressing crush injuries. Our literature review found that oil decontamination of cutaneous injuries may be best accomplished with oil-based cleansers. Certainly, this topic has significant importance for the potentially carcinogenic petroleum compounds that pervade virtually every aspect of modern human life.

## Introduction

Petroleum products in the form of transportation fuels, heating and electricity fuels, asphalt and road oil, plastics, and synthetics fuel a major global industry [1]. The US Energy Information Administration estimates that worldwide petroleum consumption will grow by 1.0 million barrels per day in 2013 with China as the leading global consumer [2]. North America is the continent currently producing the most crude oil [2]. In the US alone, the oil and gas extraction industry employs almost 200,000 employees [3].

**Figure 1:**
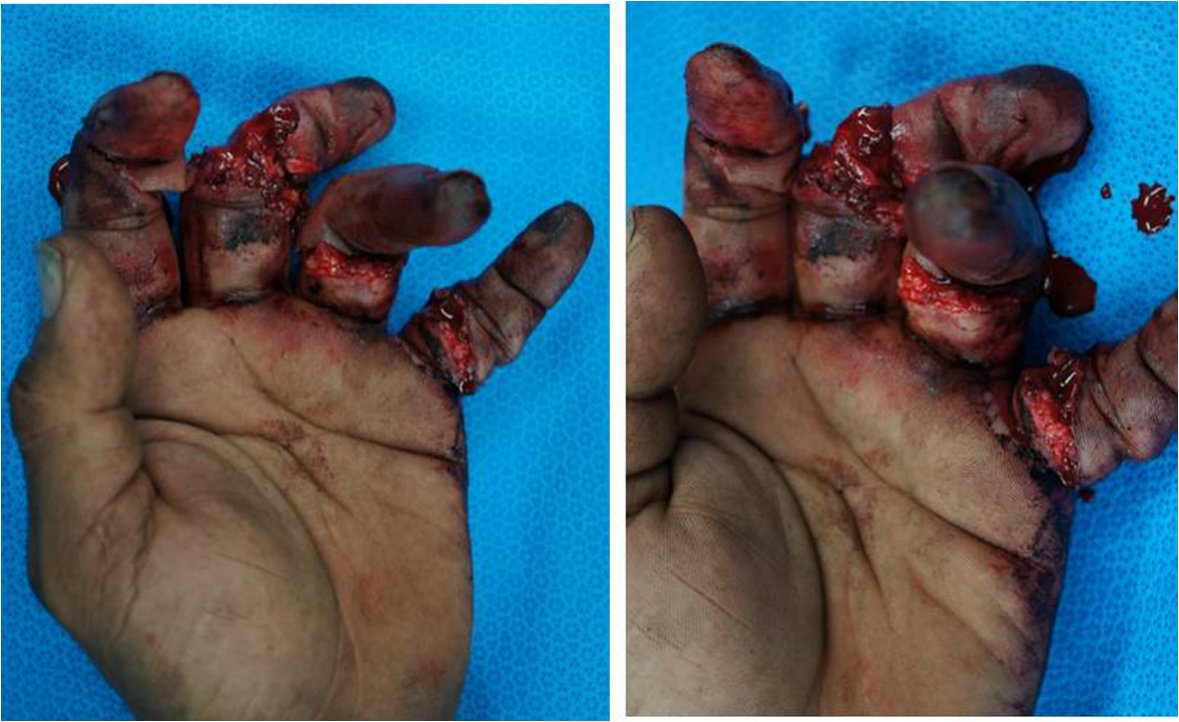
Crush injured hand with oil contamination of volar wounds involving flexor tendons, neurovascular bundles and bone.

**Figure 2:**
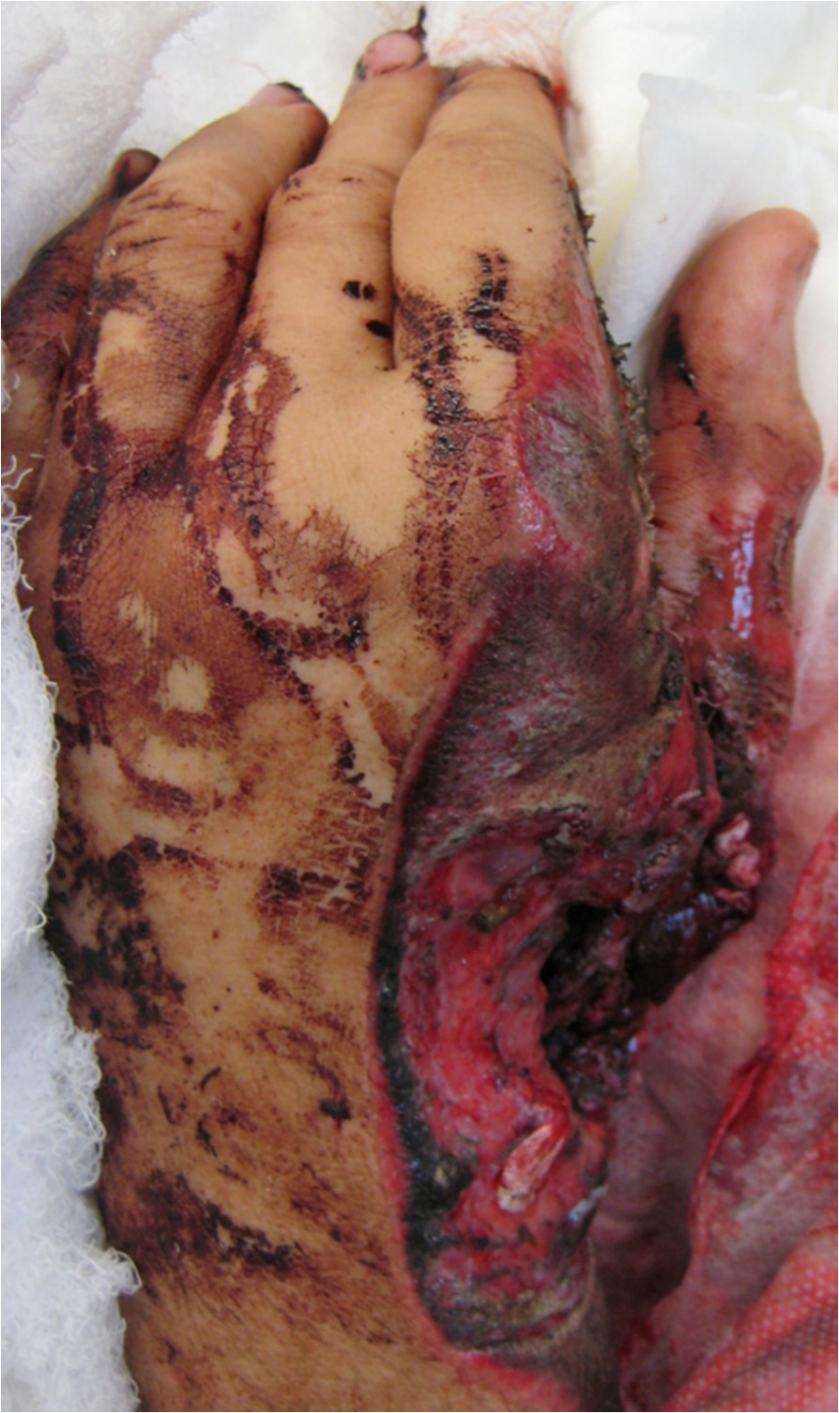
Crush amputated hand with extensive oil contamination involving all anatomic structures from the forearm to the midhand level.

Preliminary data from the Bureau of Labor and Statistics indicate an increased rate of full-time worker fatalities in the oil and gas extraction industry in 2012 of 25 per 100, compared to 12 per 100 in 2009 [3]. The most frequent causes of these fatalities were transportation incidents (41%), contact with objects and equipment (25%), and fires and explosions (15%) [4]. In addition to industrial injuries, oil-contaminated wounds are also commonly encountered in military injuries and industry-environmental clean-up, with far-reaching effects in both mammals and birds [5]. Due to work with large equipment and explosive devices, the potential for crush injuries is evident. By definition, a crush injury results from continuous prolonged pressure on a limb leading to direct and local limb injury [6]. Over the prolonged period, necrotic myocytes begin to swell leading to edema in the affected limb. Crush injuries cause significant morbidity due to compromise of vascular and neurologic supply, direct pressure, and effects of contaminants in the wound [7]. Of particular interest in the realm of crush injuries are those that involve contamination with petroleum. While surgical debridement of contaminated wounds with excision of crushed and contaminated tissues is considered standard of care for most wounds, there are functionally irreplaceable structures which cannot be injudiciously excised. Examples are oil contaminated crush injuries to the upper extremity with neurovascular bundle involvement (Figures 1 and 2). This paper surveys the literature regarding the most effective methods of oil decontamination from cutaneous wounds.

## Chemistry of oil and decontaminants

The predominant structure of petroleum is that of a carbon and hydrogen backbone which confers the universal non-polar property to all oils. Variations on this backbone include alkanes, aromatic hydrocarbons, and cycloalkanes which confer unique boiling points and potential energy properties. Geographical location and refining techniques create an even greater structural diversity of petroleum compounds. Oil’s hydrophobic nature results in immiscibility with polar compounds, such as water, and solubility with non-polar solvents. In addition, lipophilic compounds such as oil tend to penetrate the epidermis and even deeper into the skin’s intercellular lipid matrix much more readily than hydrophilic compounds [8]. Factors to be considered regarding the effectiveness of a decontaminant include the chemical properties of the contaminant, the amount of contaminant on the skin, the timing of decontamination, the duration of decontamination, and the anatomic location [8]. The simple concept of “like dissolves like” where a non-polar substance tends to maximize interactions with other non-polar substances to increase entropy is the overriding principle in oil decontamination of the skin [9].

## What does the literature say?

An extensive literature review on Pubmed and Google was performed for all articles assessing oil decontamination from cutaneous wounds with a particular focus on crush injuries. Search terms included “oil petroleum decontamination skin”, “oil petroleum decontamination cutaneous”, and “oil petroleum decontamination crush injury”. No studies to date have evaluated oil decontamination of crush injuries. While there was a great paucity of data in the literature, this expert opinion review will discuss the six most relevant and applicable studies regarding oil decontamination of cutaneous wounds.

### Case reports

One of the earliest reports about oil decontamination of a skin wound is a case report from 1967 [10]. In this report, a roofing worker presented with a severe facial tar burn to his face, eyelids, and ear after tar heated to 450°F was splashed on his face. In response to poor experience using various solvents, soaps, and detergents, the medical team decided to attempt a novel treatment with Neosporin. Neosporin ointment consists of neomycin, bacitracin, and polymixin B in a petrolatum base. The patient’s face was coated with Neosporin ointment at hourly intervals. By 12 hours, all tar was dissolved and no infection occurred. While the Neosporin antibiotics prevented infection, the successful decontamination of tar was believed to be due to the petrolatum component. Petrolatum is an oleaginous colloidal suspension of solid microcrystalline waxes in petroleum oil composed of long-chain aliphatic hydrocarbons. Long-chain hydrocarbon petrolatum allows longer contact time with the wound. Shorter-chain hydrocarbons have lower boiling points, becoming volatile more quickly and would have insufficient contact time with the tar. The principle rests on the “like dissolves like” theory in which the petrolatum-base of Neosporin “acted to dissolve the tar.” This same principle is used with oil-based cleansers, which work to remove sebum oils from the skin [11].

A similar protocol was used in a 1983 report of 42 patients treated for hot tar and asphalt injury [12]. Injuries were industry-related (roofing and road-paving) in 95% of patients, with 71.4% due to tar and 28.6% due to asphalt. Initial treatment included application of cold water to the injury in order to enhance cooling and solidification. Wounds were then treated with the “direct liberal application and gentle wiping” of a surface-active, petroleum-based solvent (De-Solt-it). This hydrocarbon solvent consists of 70% petroleum distillate, 25-27% limonene (orange oil), 2-3% lanolin, and 1% surfactant (dioctyl sulfosuccinate or DS). Following removal of the tar/asphalt from the wound, patients were treated with standard burn protocol. For 63.4% of patients, this involved surgical treatment (excision and grafting 58.5%, excision and primary closure 4.9%, multiple excisions and grafting 7.1%, late reconstructions 7.1%). Mean number of days lost from work was 46.4 and 80% of hospitalized patients returned to work in 6 weeks. Complications occurred in 14.3% of patients including donor site infection (7.1%), burn wound infection (4.8%), and pulmonary embolus (2.4%). The authors of this report maintain that the De-Solv-it solvent was non-toxic, non-irritating, and more effective at removing tar than previous agents allowing for an “aggressive, early, back-to-work philosophy”. They explain that the “ideal solvent is a chemical of close structural affinity to the solute” [12]. Commercial tars and asphalts are long-chain aliphatic and aromatic hydrocarbons which are theoretically dissolved by the long-chain petroleum distillate found in the solvent.

### Experimental studies

Decontamination of petroleum compounds was investigated in a rat model study [13]. One hundred rats were killed and then underwent a contamination protocol with refined oil, crude oil, axle grease, or roofing tar. These compounds are listed in order of increasing boiling point, molecular weight, and viscosity which, the study authors hypothesized, should make it increasingly difficult for a solvent to penetrate the contaminant. The contaminated area was then cleansed with one of five cleansing solutions: 10% DS, 1% DS, chlorhexidine surgical scrub, Goop commercial clean-up solution, or normal saline with polyethylene glycol solvent. Dioctyl sulfosuccinate is a cheap surfactant laxative and cerumenolytic found in bath products, shampoos, and skin cleansers. It is mostly insoluble in water due to its hydrophobic nature. Fluorometry was used to investigate post-cleansing petroleum residue. No significant difference was found between the 5 cleansers for refined oil. However, for crude oil, 10% DS was significantly better at removing the contaminant than chlorhexidine (p < 0.01), Goop (p < 0.05), and the solvent (p < 0.01). Similarly, for axle grease, 10% DS and 1% DS were both significantly better than chlorhexidine (p < 0.05), Goop (p < 0.001), and the solvent (p < 0.01). The results were most dramatic for the roofing tar contaminant for which 10% DS was significantly better than all other cleansers (p < 0.001). The study concluded that DS is significantly more effective at petroleum decontamination than traditionally-used surgical (chlorhexidine) and commercial (Goop) cleansers.

Another study performed in rhesus monkeys investigated in vivo decontamination of methylene diphenyl diisocyanate (MDI) [14]. This known dermal and respiratory sensitizer is partly oil-miscible, thus resembling some chemical similarities to petroleum in regards to decontamination. MDI is used most notably in the manufacture of polyurethane polymers as foams, adhesives, coatings, binders, and sealants [15]. In the study, 4 rhesus monkeys were contaminated with MDI in 24 marked sites with skin intact. Six different cleansers were evaluated for efficacy in MDI decontamination which fall into two distinct classes: aqueous washings (water only, 5% soap, 50% soap) and lipophilic washings (polypropylene glycol, a polyglycol-based cleanser termed PG-C, corn oil). Washings were performed at 5 minutes, 1 hour, 4 hours, and 8 hours in addition to tape stripping at each of these time intervals to measure radioactivity as a surrogate for MDI residue. At 4 and 8 hrs, the 3 lipophilic washes were significantly better than the 3 water-based washings at removing MDI (p < 0.05). For water-based solutions, 60-70% of MDI was removed at 5 minutes, however, by 8 hours only 29, 37, and 46% of MDI was removed by water only, 5% soap, and 50% soap, respectively. At all time periods, 68-95% of MDI was removed by the lipophilic cleansers. This study demonstrated superior decontaminating ability of non-traditional lipophilic cleansers over traditional water and soap-water formulations. It also highlighted the importance the time factor in skin decontamination as there were great differences in recovered MDI residue when cleansing was performed within the first hour compared to 4 and 8 hours later.

A similar *in vitro* study was performed to evaluate decontamination of 4 contaminants: methylenededianiline, chlorpyrophos, pentachlorphenol, and benzo-a-pyrine [16]. These contaminants are listed in order of increasing octanol/water solubility, representing the spectrum of hydrophilic to lipophilic. The study authors state that the optimal decontaminant will “promote skin’s barrier function while optimizing contaminant’s removal based on its solubility in the decontamination solvent” [16]. Each contaminant was cleansed with one of four decontaminants: water, 10% ivory soap, a polyethylene glycol-based cleanser, and an oil-based cleanser. For the most lipophilic contaminant, benzo-a-pyrene, the oil-based cleanser was superior to the other three tested cleansers in its ability to dissolve the contaminant. Study authors recommend choosing the most effective decontaminant based on the contaminant’s partition coefficient. For lipophilic contaminants including TDI, benzo-a-pyridine, MDI, pentachlorphenol, parathion, PCB, DDT, diesel oil, toluene, and benzene, an oil-based cleanser is recommended. Water and soap may be appropriate for hydrophilic contaminants such as formaldehyde, propanol, ethyl carbonate, and hydrazine.

While the above studies promote the novel use of petroleum or oil-based cleansers as the most effective methods for oil decontamination of the skin, a 1990 rat model study warned about the toxicity of these agents [17]. Three cleansing agents were evaluated: liquid dishwashing detergent (soap) and water, Boraxo degreaser, and Go-Jo degreaser. Wounds made in the rats were treated by closure alone (control) or closure after application of Lubrimatic grease, soap and water, Boraxo, Go-Jo, or a combination of grease and each of the 3 cleansing agents. Histological scoring of the wounds looked for inflammation, fibrosis, surface ulceration, and crust formation. Results indicated that wounds treated with Boraxo or Go-Jo degreasers had the highest number adverse tissue reactions at 1 week (p < 0.001). Grease alone, soap and water, and grease with soap and water showed similar adverse reactions to the control. Petroleum-based degreasing cleaners in open grease-contaminated wounds resulted in an increased inflammatory response (adverse tissue reactions) compared to grease alone and a delay in wound healing. The study authors concluded that soap and water was best for removing grease from open wounds and warned that degreasing cleaners should only be used on intact skin, not on open wounds. The authors also warn that case reports promoting the effectiveness of petroleum-based cleansers should not take the place of “experimental research” [18].

## Conclusion

Modern human life depends on petroleum. The petroleum industry is vast, involving large machinery and dangerous working conditions. Oil contaminated crush injuries are one of many possible injuries, some fatal, associated with the production and manufacturing of oil. This review addresses a significant and under-examined topic that has both clinical and environmental impact. While there were no models specifically investigating crush injuries, we present important case reports and animal model studies that explore the most effective solvent for oil decontamination from cutaneous wounds. Most studies recommend a non-polar solvent such as petroleum oil-based mixtures or the waxy solid, dioctyl sulfosuccinate. For healthcare professionals in clinical practice, use of a non-polar solvent to cleanse wounds regardless of debridement may be beneficial to preserve irreplaceable structures.

Certainly, the field would greatly benefit from additional case reports and anecdotal experiences with the ultimate goal of large, randomized controlled trials to rigorously determine a treatment advantage. However, this topic is particularly prone to ethical limitations of randomized trials as the apparent reason for the paucity of experimental studies in humans is the potentially fatal health effect of petroleum. According to the Center for Disease Control, petroleum compounds can affect the human central nervous system including peripheral neuropathy and paralysis, as well as the blood, immune system, liver, spleen, kidneys, lungs, and a developing fetus [19]. In addition, benzene, a group 1 carcinogen, has been linked to leukemia, and other petroleum products are classified as group 2 carcinogens [20]. Certainly, the toxic properties of a chemical that is used in almost every aspect of modern society highlights even more the importance of an effective decontamination protocol for petroleum.

## Abbreviations

DS: Dioctyl sulfosuccinate; MDI: Methylene diphenyl diisocyanate; PG-C: Polyglycol-based cleanser; TDI: Toluene diisocyante; PCB: Polychlorinated biphenyl; DDT: Dichloro-diphenyl-trichloroethane.

## Competing interests

The authors have no personal or financial conflicts to declare.

## Author contributions

KI and RD were responsible for study design. CK and MA performed the literature search. CK performed the data collection, data analysis, data interpretation, and writing of the manuscript. MA, KI, and RD performed critical revision of the manuscript. All authors read and approved the final manuscript.
